# Treatment of Femoral Neck Fractures with Cannulated Screw Invasive Internal Fixation Assisted by Orthopaedic Surgery Robot Positioning System

**DOI:** 10.1111/os.12548

**Published:** 2019-10-29

**Authors:** Xiao‐dong Wang, Hai Lan, Kai‐Nan Li

**Affiliations:** ^1^ Zunyi Medical University Zunyi, Guizhou China; ^2^ Department of Orthopaedics Affiliated Hospital of Chengdu University Chengdu, Sichuan China

**Keywords:** Orthopaedic surgery robot positioning system, Femoral neck fractures, Cannulated screw internal fixation

## Abstract

**Objective:**

To investigate the clinical efficacy and advantages of cannulated screw internal fixation assisted by the orthopaedic surgery robot positioning system in the treatment of femoral neck fractures.

**Methods:**

The clinical data of 128 patients with femoral neck fractures which had been treated with cannulated screw internal fixation from January 2016 to July 2018 were retrospectively analyzed. Among them, 63 patients were treated with cannulated screw assisted by orthopedic robot positioning system (orthopaedic surgery robot group), and 65 patients were treated with traditional cannulated screw (traditional surgery group). The operation time, number of intraoperative fluoroscopy, number of guide needle placements, and the amount of operative blood loss were compared between the two groups. The success rate of one‐time nail placement and the fracture healing rate were calculated. Fracture healing and internal fixation were observed. The hip joint function was evaluated by the Harris hip score 1 year after operation.

**Results:**

All patients were followed up for 12 to 24 months. The operation time was 65.70 ± 9.87 min in the robot group and 73.74 ± 9.78 min in the traditional group. The number of intraoperative fluoroscopy was 13.67 ± 4.39 times in the robot group and 17.09 ± 4.02 times in the traditional group. The number of guide needle placements was 9.95 ± 3.72 times in the robot group and 13.78 ± 4.39 times in the traditional surgery group. The success rate of one‐time nail placement was 100% (63/63) in the robot group and 49.23% (32/65) in the traditional group. The amount of operative blood loss was 15.25 ± 6.21 mL in the robot group and 25.51 ± 6.97 mL in the traditional group. Compared with the traditional group, the robot group had shorter operation time, less fluoroscopy, less needle placement, less bleeding, and higher success rate of one‐time nail placement. There was a significant difference between the two groups (*P* < 0.05). In the robot group, there was no infection, loosening of internal fixation, fracture displacement, and osteonecrosis of femoral head during the follow‐up period. The fracture healing rate was 100% (63/63). In the traditional group, there were two cases of loosening of internal fixation and one case of osteonecrosis of femoral head during the follow‐up period. The fracture healing rate was 100% (65/65). All patients were evaluated for hip joint function 1 year after operation. The Harris hip score in the robot group was 86.86 ± 4.74, and the Harris hip score in the traditional surgery group was 83.08 ± 5.44. Compared with the traditional group, the Harris hip score in the robot group was higher than that in the traditional group. There was significant difference between the two groups (*P* < 0.05). The excellent and good rate were 92.06% (58/63) in the robot group and 80% (52/65) in the traditional group. There was no significant difference between the two groups (*P* > 0.05).

**Conclusion:**

Cannulated screw internal fixation assisted by the orthopaedic surgery robot positioning system is an ideal method for the treatment of femoral neck fractures. This method has the advantages of relatively simple operation, more accurate screw placement during operation, high success rate of one‐time nail placement, short operation time, less surgical trauma, less radiation, and good recovery of hip function.

## Introduction

Medical robotics is a new cross‐research field which integrates medicine, biomechanics, mechanical mechanics, material science, computer science, robotics, and so on. In 1985, Kwoh *et al*. applied industrial robot, Puma200, to neurosurgery for the first time^1^. Medical robotics can provide adequate support for doctors' decision‐making and operation in visual, tactile and auditory aspects, and expand doctors' operating skills. It can effectively improve the quality of surgical diagnosis and evaluation, target localization, precision operation, and surgical operation. Medical robot technology is used more and more in clinical treatment in various departments. In 1991, the world's first orthopaedic robot, RoboDoc, was born and completed a clinical trial in July of that year. In 1992, the first case of the total hip replacement was completed^2^. With the improvement of medical technology and the rapid development of minimally invasive surgery, the various functions of the orthopaedic surgery robot positioning system continues to develop and improve, at home and abroad. Robot‐assisted orthopaedic surgery is also accepted by more and more surgeons. The orthopaedic surgery robot positioning system uses the function of computer data processing to analyze and process the patient's image data obtained from X‐ray, CT, and other imaging equipment^3^, ^4^. At the same time, with the help of external spatial coordinates tracking equipment, it measures the spatial coordinates of the patient's surgical target area and the surgical instrument or robot in order to obtain the relative positional relationship between them^5^. This guides the doctor to carry out accurate, rapid, and safe positioning and implantation of plants^6^. With its significant advantages in improving the accuracy of surgery, reducing surgical trauma and intraoperative radiation damage, and increasing the success rate of the operation, the orthopaedic surgery robot positioning system has shown excellent clinical application value and has received more and more attention.

Hip fracture is associated with limited movement, chronic pain and disability, loss of independence of life, and decline in quality of life. Twenty per cent to 30% of patients with hip fractures die within a year^7^, ^8^. Femoral neck fractures are the most common type of hip fracture. With the aggravation of the aging of the world population, the incidence rate is increasing year by year^9^. Femoral neck fractures can occur in any age group, especially in the middle‐aged and elderly. Due to high‐energy trauma, young adults can also suffer femoral neck fractures. Complications such as fracture nonunion and osteonecrosis of femoral head easily occur after the fracture of the femoral neck^10^. The Garden classification of femoral neck fractures divides these fractures into four types^11^. Type I is an incomplete fracture and the bone's trabeculae below the femoral neck is intact. This type includes the so‐called “abduction embedded fracture.” Type II is a complete fracture, but there is no displacement. Type III is a complete fracture with partial displacement. The distal displacement and external rotation of the fracture can be seen on the X‐ray film of this type of fracture. The femoral head is often tilted backward, and there is still some contact at the end of the fracture. Type IV is a complete fracture with complete displacement. The X‐ray film of this type of fracture showed that there was no contact at the broken end of the fracture. The relative relationship between the femoral head and the acetabulum is normal. The treatment of femoral neck fractures can be divided into conservative treatment and surgical treatment. Because conservative treatment requires the patient to stay in bed for a long time, the incidence of complications in conservative treatment, such as pulmonary infection and thrombosis, is high. Therefore, most scholars believe that surgical treatment should be the first choice for patients with femoral neck fractures^12^. Patients with femoral neck fractures who have no concomitant disease of the hip joint can obtain good reduction and fixation and can tolerate the operation. These include Garden type I and type II fractures, Garden type III and type IV under 65 years old and there was no hip osteoarthritis and femoral head necrosis before fracture. Displaced fracture, old age, and poor general condition, combined with important organ dysfunction, cannot tolerate joint replacement surgery. At present, the commonly used surgical treatment is closed reduction and cannulated screw internal fixation through traction. However, it is difficult to ensure that each screw is in the best position during surgery. Secondary nail placement is more common. The risk of postoperative internal fixation instability and fracture nonunion is still high. Schep *et al*. found that the position and direction of the screws during operation were closely related to fracture stability and fracture healing^13^. Accurate screw placement can increase the stability of internal fixation of femoral neck fractures and reduce the risk of fracture nonunion^14^. The orthopaedic surgery robot positioning system technique was used to assist the operation of cannulated screw internal fixation of femoral neck fractures. It is helpful to accurately locate the placement direction of the screw guide needle, improve the success rate of one‐time screw placement, shorten the operation time, and reduce the surgical trauma. This method can effectively reduce the injury of patients and health care workers caused by X‐ray. It can also promote the rapid recovery of patients after operation.

The purpose of this study is as follows: (i) to compare the clinical efficacy of cannulated screw internal fixation assisted by the orthopaedic surgery robot positioning system and traditional cannulated screw internal fixation in the treatment of femoral neck fractures; and (ii) to investigate the advantages of cannulated screw internal fixation assisted by the orthopaedic surgery robot positioning system in the treatment of femoral neck fractures.

## Materials and Methods

### *Inclusion and Exclusion Criteria*

#### *Inclusion Criteria*

The inclusion criteria included: (i) patients younger than 65 years old and diagnosed with unilateral closed femoral neck fractures by X‐ray or CT; (ii) The surgical methods are based on closed reduction and use orthopaedic surgery robot positioning system to assist cannulated screw internal fixation in the treatment of femoral neck fractures or traditional cannulated screw internal fixation in the treatment of femoral neck fractures; (iii) the main evaluation indicators included the operation time, the number of intraoperative fluoroscopy, the number of guide needle placements, the amount of operative blood loss, the success rate of one‐time nail placement, the fracture healing rate, the Harris hip score, and the excellent and good rate of hip joint function; and (iv) the study was a retrospective case–control study.

### *Exclusion Criteria*

Exclusion criteria included: (i) patients had a history of hip fracture on the affected side; (ii) fracture belongs to pathological fracture, such as bone metastasis of cancer, primary bone tumor, metabolic bone disease, and so on; (iii) the affected hip has moderate to severe hip arthritis or osteonecrosis of femoral head; and (iv) the postoperative follow‐up period was less than 1 year.

### *General Information of Participants*

The clinical data of 128 patients with femoral neck fractures which had been treated with cannulated screw internal fixation from January 2016 to July 2018 were retrospectively analyzed. According to surgical methods, patients were divided into two groups for comparison. Among them, 63 patients underwent internal fixation with cannulated screw assisted by robot navigation in orthopaedic surgery, and 65 patients underwent internal fixation with traditional cannulated screw. All patients signed informed consent for surgery.

In the orthopaedic surgery robot group, 63 patients (30 males and 33 females) were aged from 25 to 64 years, with an average age of 49.03 years. In the traditional surgery group, 65 patients (31 males and 34 females) were aged from 29 to 64 years, with an average age of 49.80 years.

Patient characteristics are shown in Table 1. There were no statistical differences in gender, age, injury side, and fracture type between the two groups, and they were comparable (*P* > 0.05).

**Table 1:** Patient characteristics of the two groups

Groups	The number of cases	Age (years)	Gender (cases)		Injury side (cases)		Garden classification (cases)			
			Male	Female	Left	Right	I	II	III	IV
Orthopaedic surgery robot group	63	49.03 ± 8.23	30	33	25	38	15	34	10	4
Traditional surgery group	65	49.80 ± 7.68	31	34	29	36	14	36	10	5
*P*‐value		0.586^a^ *	1.000^b^ *		0.596^b^ *		0.991^b^ *			

a *t*‐test

b Pearson chi‐square test

* No statistically significant.

Internally fixed implants used association for the study of osteosynthesis (AO) (7.3 mm) cannulated screw produced by DePuy Synthes Company, Switzerland.

**Figure 1:**
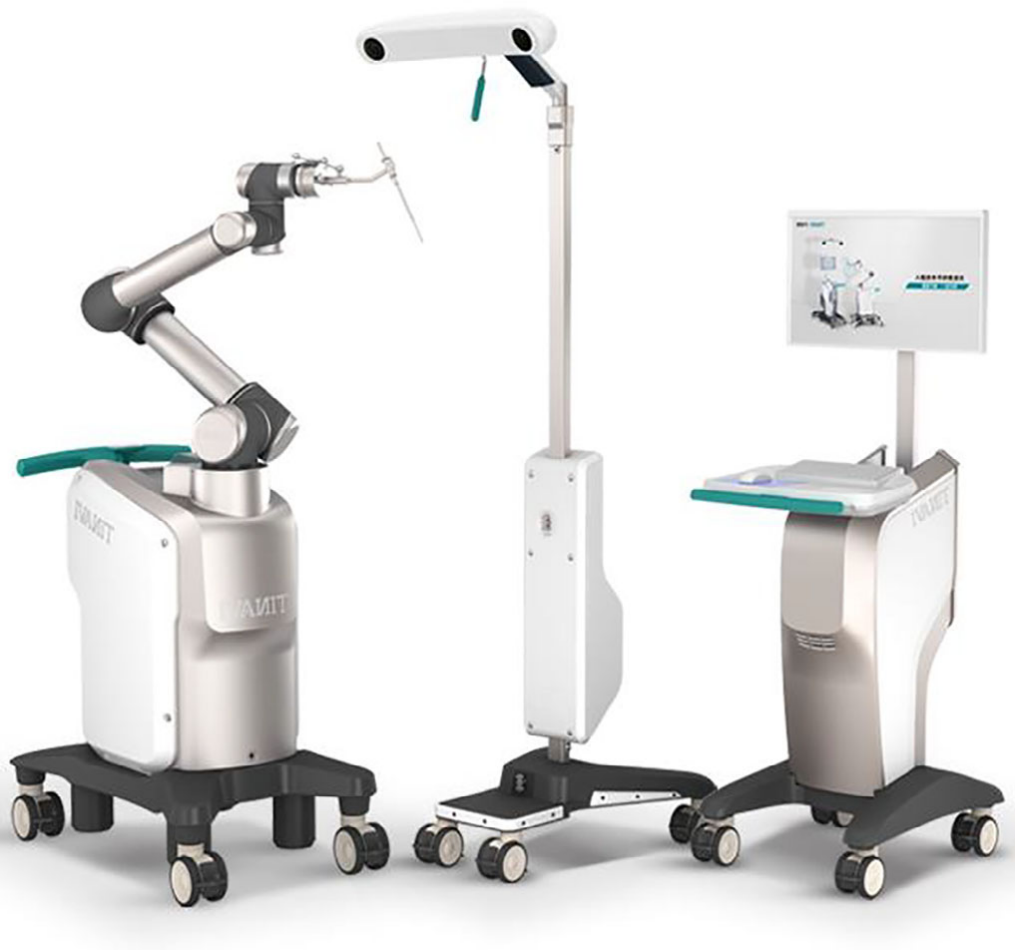
TiRobot, mainly composed of a workstation, an optical tracking system, and a robotic arm (Photo provided by Beijing Tianzhihang Medical Technology).

In the orthopaedic surgery robot group, the operation was performed with the help of TiRobot, the third generation of the orthopaedic surgery robot of Beijing Tianzhihang Medical Technology (Beijing, China) (Fig. 1).

### *Surgical Methods*

Cannulated Screw Internal Fixation Assisted by the Orthopaedic Surgery Robot Positioning System

#### *Robot Preoperative Preparation*

The operation was performed with the assistance of the orthopaedic surgery robot TiRobot^15^. Check whether the robot is fully equipped before operation. Conduct routine preoperative preparation for the workstation, the optical tracking system, the robotic arm, the C‐arm X‐ray machine, and other equipment. Turn on the power and connect the equipment. Check that the device is functioning properly. Log in to the system, record medical records, and select surgical tools.

#### *Patient Preparation*

All patients in the group underwent surgery under general anesthesia. After successful anesthesia, the patient lay on his or her back on the orthopaedic traction bed. The pelvis maintains a horizontal position. The affected limb was continuously pulled and fixed. In order to prevent the movement of lower limb position during operation, the healthy lower limb maintained a certain intensity of traction at the same time. Closed reduction of fracture by manual reduction and adjustment of traction was conducted. The C‐arm X‐ray machine was used to examine the affected hip joint to confirm that the reduction of femoral neck fractures were satisfactory.

#### *Disinfection and Installation of the Positioning Ruler*

Routine surgical disinfection of femoral neck fractures and spread aseptic cloth. An anchor nail was placed in the anterior superior iliac spine on the affected side and an optical tracer was installed. The positioning ruler is firmly assembled with the robotic arm. Operate the workstation for ruler calibration. Adjust the positioning ruler to the appropriate position.

#### *Intraoperative Image Acquisition*

**Figure 2:**
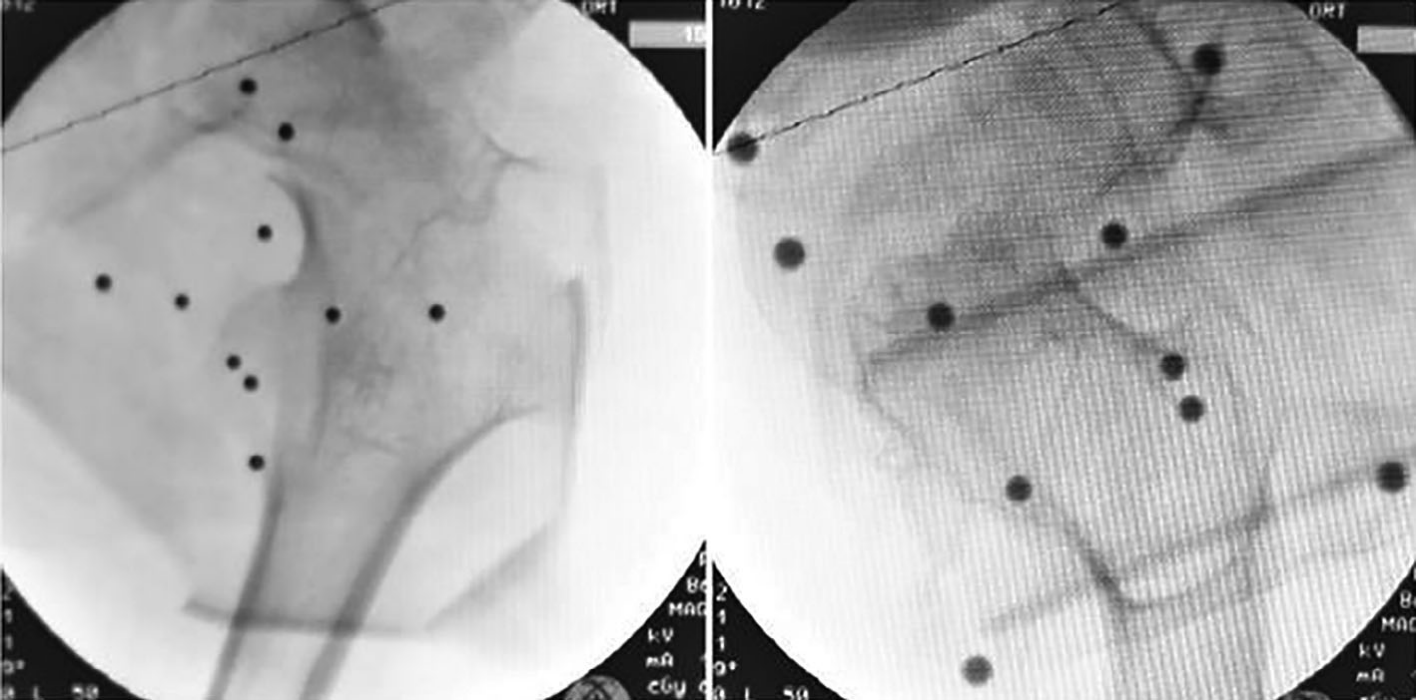
Anteroposterior and lateral images of the hip joint, with all 10 positioning points on the positioning ruler in it.

After the orthopaedic surgery robot was placed successfully, the positive and lateral images of the affected hip joint were collected by the C‐arm X‐ray machine. At the same time, all the 10 positioning points on the positioning ruler were included in the positive and lateral image field of view (Fig. 2). The positive and lateral images collected by the C‐arm X‐ray machine were imported into the workstation.

#### *Path Planning*

**Figure 3:**
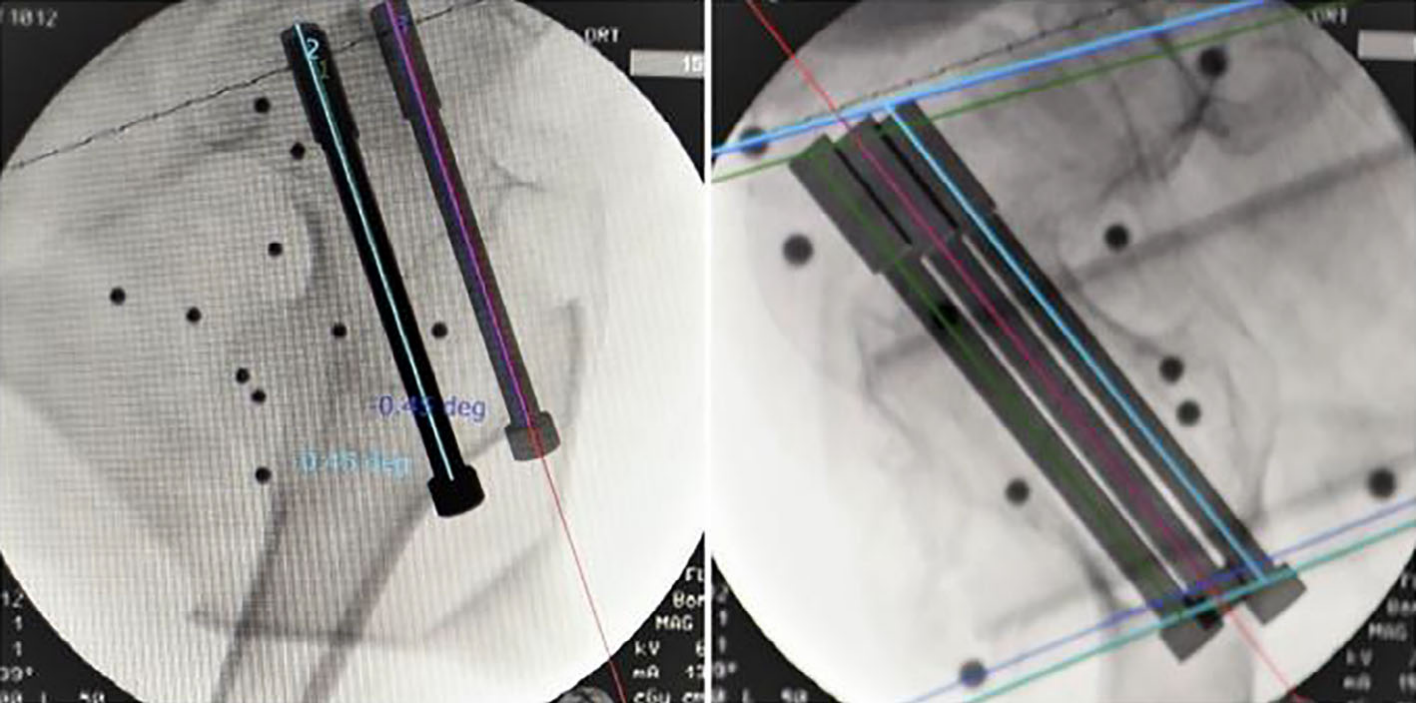
Anteroposterior and lateral images of the hip joint, which was imported into the workstation. Design the placement planning path and simulation graph of the screw in the workstation.

Design the placement planning path and simulation graph of the screw in the workstation (Fig. 3). The planned three screws follow the principle of parallel dispersion and show an “inverted triangle” layout. The tip of the needle should be located under the cartilage of the femoral head to 0.5 mm. Complete surgical planning of screw placement position, direction, and depth. The reference value of screw length is calculated automatically by the system.

#### *Guide Needle Placement*

**Figure 4:**
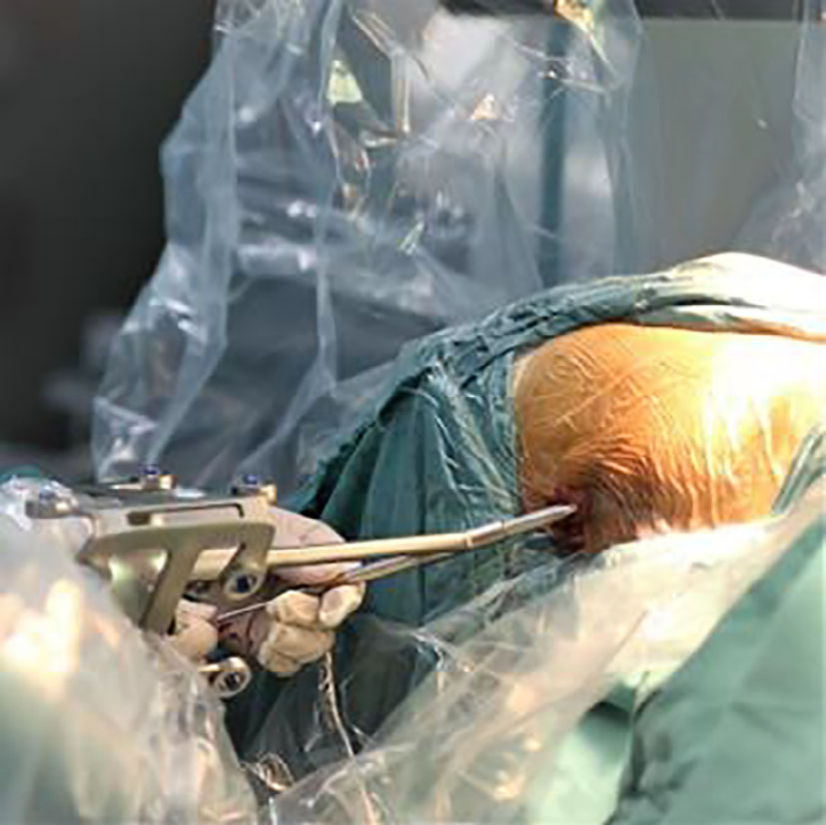
Photograph of the operative region of a patient's hip. With the assistance of the robot arm, the special guidance needle configured by the robot is inserted through the skin.

**Figure 5:**
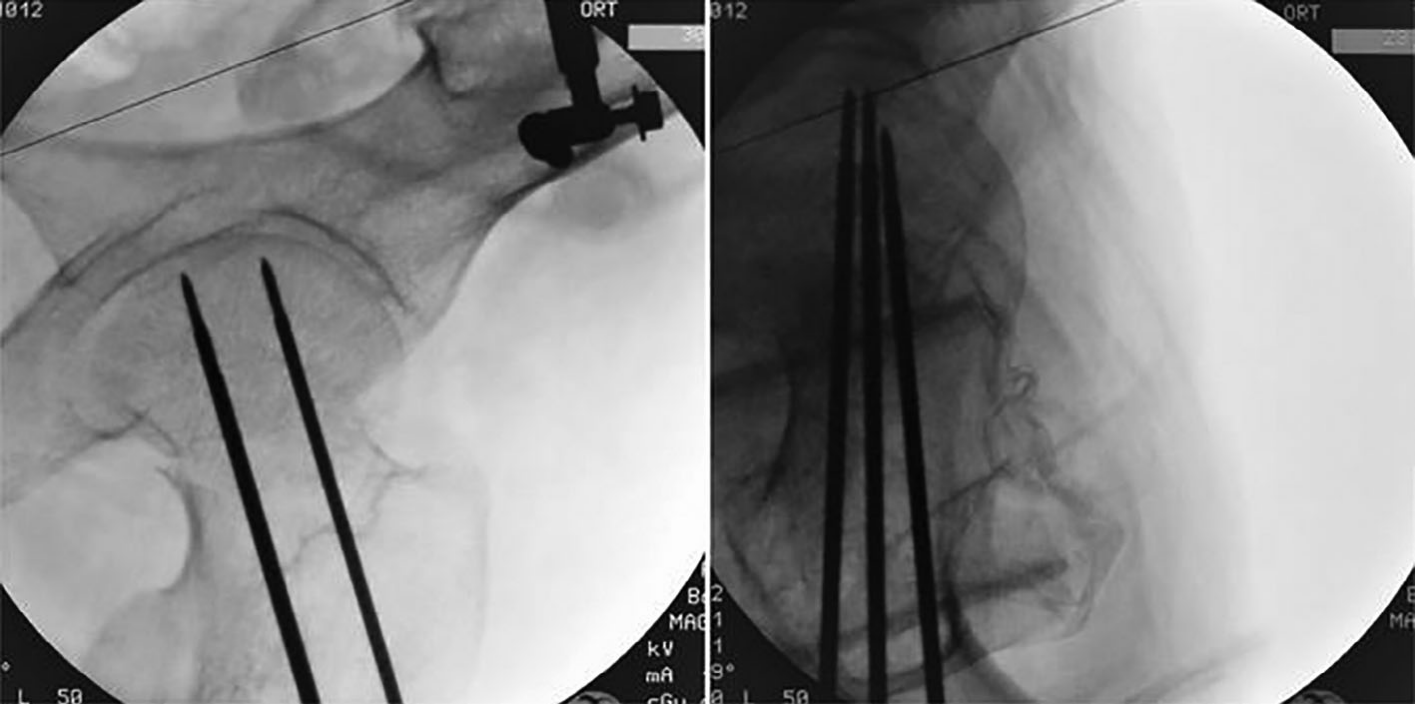
Anteroposterior and lateral images of the hip joint after insertion of three guides.

Run the robotic arm. Navigate according to the planned path to locate the placement direction and entry point of each guide needle. A special guide needle configured by the robot is inserted into the skin (Fig. 4). The C‐arm fluoroscopy of the affected hip joint is carried out in the positive and lateral position. The position, angle, and depth of the three guide needles were observed (Fig. 5). Taking the calculated length of the system as a reference, the needle length is measured with a special bathymetric ruler in the process of needle injection.

#### *Cannulated Screw Placement*

Find the satisfactory position using the routine cannulated screw placement procedure. Expand the holes along the three guide needles. Place cannulated screws in turn and remove the guide needle. The placement order of cannulated screws is as follows: lower screws, front screws, rear screws. The incision was sutured after the fluoroscopy was correct again.

### *Traditional Cannulated Screw Internal Fixation*

#### *Patient Preparation*

Compared with the orthopaedic surgery robot group, all patients in traditional surgery group had the same standards of anesthesia, posture, and fracture reduction. The C‐arm X‐ray machine was used to examine the affected hip joint to confirm that the reduction of the femoral neck fractures were satisfactory.

#### *Guide Needle Placement*

About 3 cm of the femur was taken below the lateral greater trochanter to make a longitudinal incision extending to the distal end of the 6 cm. Incision of skin, subcutaneous tissue and fascia lata, pure separation of lateral thigh muscle, exposure of the lateral wall of the femur. Three guide needles were drilled in the direction of the neck of the thigh, which followed the principle of parallel dispersion and showed an “inverted triangle” layout. C‐arm fluoroscopy of the affected hip joint was carried out in the positive and lateral position. The position, angle, and depth of the three guide needles were observed. The tip of the needle should be located under the cartilage of the femoral head to 0.5 mm. If the position is unsatisfactory, pull out the guide needle to adjust the direction and redrill in.

#### *Cannulated Screw Placement*

After the position was satisfied, the injection lengths of three guide needles were measured manually. The holes were expanded along the three guide needles. Place cannulated screws in turn and remove the guide needle. The placement order of cannulated screws is as follows: lower screws, front screws, rear screws. The incision was sutured after the fluoroscopy was correct again.

### *Observation Indicators*

#### *Operation Time*

The operation time began with the aseptic operation sheet and ended at the end of the suture incision. The operation time was mainly affected by the number of intraoperative fluoroscopy. Repeated intraoperative fluoroscopy can prolong the operation time.

#### *Number of Intraoperative Fluoroscopy*

The number of perspective images taken during the operation was recorded. This includes a perspective image of each positive and lateral position. Repeated puncture of screw guide needles will increase the number of intraoperative fluoroscopy.

#### *Number of Guide Needle Placements*

The number of screw guide needle placements was recorded. The successful placement of cannulated screws requires the guidance of screw guide needles. Whether the screw guide needle can be successfully placed in the exact position at one time will affect the operation time and the number of intraoperative fluoroscopy. After recording the times of the screw guide needle placement, the success rate of one‐time nail placement was calculated.

#### *Amount of Operative Blood Loss*

The blood was collected by the drainage bag. Prolonging the duration of the operation will increase the operative blood loss.

#### *Fracture Healing*

All patients were followed up regularly for 12 to 24 months. The positive and lateral X‐ray films of the affected hip joint were reexamined. Fracture healing and internal fixation were observed, such as whether the cannulated screw is stable, whether the fracture line disappears, whether the broken end of the fracture is displaced, and so on. At the same time, the recovery of hip joint function was observed. The fracture healing rate was calculated.

#### *Harris Hip Score*

The functional recovery of hip joint after operation was evaluated by the Harris hip score^16^. All patients were evaluated for hip joint function 1 year after operation. The hip joint function of all patients was evaluated according to the Harris hip scoring standard. It includes four aspects: pain, function, degree of deformity, and range of motion of joint. At the same time, the clinical curative effect was graded according to Harris hip score: excellent, 90 to 100; good, 80 to 89; pass, 70 to 79; and poor, <70. The excellent and good rates of hip joint function were calculated.

### *Statistical Analysis*

Statistical software IBM SPSS 20.0 (International Business Machines Corporation, Armonk, New York, USA) was used for statistical analysis. The quantitative data included the operation time, the number of intraoperative fluoroscopy, the number of guide needle placements, the amount of operative blood loss, and the Harris hip score. The measurement data between groups were tested by K‐S normal distribution test and variance homogeneity test. If the data conformed to the normal distribution and the variance was neat, the measurement data between groups were compared by t‐test for statistical analysis. All of them were expressed as mean ± standard deviation. The counting data included the success rate of one‐time nail placement and the excellent and good rate of hip joint function. The counting data between groups were compared by the Fisher exact probability method and χ^2−^test for statistical analysis. *P* < 0.05 was considered statistically significant.

## Results

The operation time, the number of intraoperative fluoroscopy, the number of guide needle placements, the amount of operative blood loss, and the Harris hip score were in accordance with normal distribution in the two groups. The intraoperative results are shown in Table 2.

**Table 2:** Comparison of the intraoperative information between the two groups (mean ± standard deviation)

Groups	Number of cases	Operation time (min)	Number of intraoperative fluoroscopy	Number of guide needle placement	Amount of operative blood loss (mL)
Orthopaedic surgery robot group	63	65.70 ± 9.87	13.67 ± 4.39	9.95 ± 3.72	15.25 ± 6.21
Traditional surgery group	65	73.74 ± 9.78	17.09 ± 4.02	13.78 ± 4.39	25.51 ± 6.97
*t*‐value		−4.628	−4.608	−5.320	−8.783
*P*‐value		<0.001*	<0.001*	<0.001*	<0.001*

* Statistically significant.

#### *Operation Time*

The operation time was 65.70 ± 9.87 min in the orthopaedic surgery robot group and 73.74 ± 9.78 min in the traditional surgery group. Compared with the traditional surgery group, the operation time of the orthopaedic surgery robot group was shortened by 10.90%. There was significant difference between the two groups (*P* < 0.05).

#### *Number of Intraoperative Fluoroscopy*

The number of intraoperative fluoroscopy was 13.67 ± 4.39 times in the orthopaedic surgery robot group and 17.09 ± 4.02 times in the traditional surgery group. Compared with the traditional surgery group, the number of intraoperative fluoroscopy in the orthopaedic surgery robot group was reduced by 20.01%. There was significant difference between the two groups (*P* < 0.05).

#### *Number of Guide Needle Placements*

The number of guide needle placements was 9.95 ± 3.72 times in orthopaedic surgery robot group and 13.78 ± 4.39 times in traditional surgery group. Compared with the traditional surgery group, the number of guide needle placements in the orthopaedic surgery robot group was reduced by 27.79%. There was significant difference between the two groups (*P* < 0.05). The success rate of one‐time nail placement was 100% (63/63) in the orthopaedic surgery robot group and 49.23% (32/65) in the traditional surgery group. The success rate of one‐time nail placement in the orthopaedic surgery robot group was 2.03 times higher than in the traditional surgery group. There was significant difference between the two groups (*P* < 0.05).

#### *Amount of Operative Blood Loss*

The amount of operative blood loss was 15.25 ± 6.21 mL in the orthopaedic surgery robot group and 25.51 ± 6.97 mL in the traditional surgery group. Compared with the traditional surgery group, the amount of operative blood loss in the orthopaedic surgery robot group was reduced by 40.22%. There was significant difference between the two groups (*P* < 0.05).

#### *Fracture Healing*

All patients were followed up for 12 to 24 months. In the orthopaedic surgery robot group, there was no infection, no loosening of internal fixation, no fracture displacement, and no osteonecrosis of femoral head during the follow‐up period. The fracture healing rate was 100% (63/63). In the traditional surgery group, there were two cases of loosening of internal fixation and one case of osteonecrosis of femoral head during the follow‐up period. The fracture healing rate was 100% (65/65).

#### *Harris Hip Score*

All patients were evaluated for hip joint function 1 year after operation. The Harris hip score was 86.86 ± 4.74 in the orthopaedic surgery robot group, compared to 83.08 ± 5.44 in the traditional surgery group. There was significant difference between the two groups (*P* < 0.05).

The clinical curative effect was graded according to the Harris hip score. In the orthopaedic surgery robot group, the clinical curative effect was excellent in 20 cases, good in 38 cases, pass in five cases, and poor in zero cases. The excellent and good rate was 92.06% (58/63). In the traditional surgery group, the clinical curative effect was excellent in five cases, good in 47 cases, pass in 11 cases, and poor in two case. The excellent and good rate was 80% (52/65). There was no significant difference between the two groups (*P* > 0.05).

## Discussion

With the aggravation of the aging of the world population, the incidence of femoral neck fractures in the middle and elderly is increasing year by year. At the same time, due to traffic accidents, high‐energy trauma, and other factors, young and middle‐aged femoral neck fractures are also increasing year by year. For the vast majority of fresh femoral neck fractures, anatomical reduction is considered first, and reliable internal fixation is used^17^. After successful closed reduction of femoral neck fractures, three cannulated screws were fixed in the “inverted triangle” layout according to the principle of parallel dispersion. This is so that the broken surface of the bone can be uniformly subjected to force, has enough stability, and the fixation strength is maximized. It is a commonly used surgical method for femoral neck fractures at present^18^, ^19^. The placement direction and position of cannulated screw during operation are closely related to whether the fracture is re‐displaced after operation, whether the fracture fixation is stable, and whether the fracture is healed or not. The more accurate placement of the cannulated screw, the higher the stability of internal fixation of femoral neck fractures and the lower the risk of fracture nonunion^20^. Therefore, the accurate selection of screw placement point and the placement of the best position are the key to the success of the operation. Traditional cannulated screw internal fixation is often influenced by the experience of surgeons. Due to the deviation of human visual acuity, the operation with bare hands is unstable. It is difficult to ensure that the position and angle of each screw are ideal. Repeatedly adjusting the screw guide pin placement path will increase the number of boreholes. This can cause muscle, soft tissue and bone damage, and increase the degree of surgical trauma and the amount of blood loss. It also prolongs the operation time, and the times of fluoroscopy were increased. This increases the amount of time patients and health care workers are exposed to radiation. It can have a bad effect on the health of both doctors and patients.

With the continuous development and functional improvement of various orthopaedic surgery robot systems at home and abroad, robot‐assisted orthopaedic surgery has been accepted by more and more surgeons. It has been widely used in clinical practice. Orthopaedic surgery robots have a wide range of applications. They are compatible with a variety of surgical planning models, have flexible movement, and can conduct a wide range of work. They are widely used in accurate localization of orthopaedic surgery. The utility model can realize the special purpose of one machine and multi‐use of one machine. Orthopaedic surgery robots can simplify the operation steps and make the operation process run more smoothly. The operation of orthopaedic surgery robots is guided by special operation software to standardize the operation. The workstation can plan multiple screw paths at a time. The system is registered automatically and can also be fine‐tuned manually. During the operation, there is multi‐dimensional image guidance, which enhances the ability of intraoperative monitoring. The robotic arm holds the device stable and reduces the fatigue of the doctor holding the device for a long time. The orthopaedic surgery robot has the function of accurate navigation and positioning, and the accuracy is up to the millimeter level. Especially for minimally invasive surgery, high‐risk areas have obvious advantages. They can effectively reduce the risk of operation and reduce the complications of operation. The robot monitors the movement of surgical patients in real time and corrects its own path. The robotic arm can move accurately to the planned position to ensure that the surgical path is consistent with the planned path. Based on the operation flow of orthopaedic surgery robots, it is only necessary to obtain images before operation, after positioning the guide needle, and after screw entry. This means that the total amount of X‐ray radiation decreases greatly. The damage caused by X‐ray radiation to patients and medical staff is therefore alleviated, which can play a certain protective role for patients and medical staff. With the application of orthopaedic surgery robot, the doctor dominates the operation planning and operation, controls the position of the implantation, and reflects the intention of the operation. The robot adopts the combination mode of passive motion and active motion to ensure accuracy and efficiency. A doctor‐led, robot‐assisted collaboration ensures the success of the operation. At the same time, the orthopaedic surgery robot adopts certain protective measures. The robot adopts motion simulation technology and joint force control technology. It can predict the trajectory or stop automatically when it encounters an obstacle, avoiding collision with patients or health care workers. Using the orthopaedic surgery robot positioning system for internal fixation surgery for fractures can obtain the best surgical path, the highest surgical efficiency and surgical accuracy^21^. This method can achieve the best results from the operation, so that patients only suffer from minor surgical injuries.

The main results of this control study are summarized as follows. First, in the study of the operation time, the number of intraoperative fluoroscopy, the number of guide needle placements, and the amount of operative blood loss in the two groups. Data of orthopaedic surgery robot group was lower than the traditional surgery group. There was significant difference between the two groups (*P* < 0.05). Second, in the study of the success rate of one‐time nail placement and the Harris hip score 1 year after the operation in the two groups. Data of orthopaedic surgery robot group was higher than the traditional surgery group. There was significant difference between the two groups (*P* < 0.05). Third, all the patients in the orthopaedic surgery robot group showed that all the three cannulated screws could achieve the fixation effect of scattered parallel and “inverted triangle” layout. The fractures healed in one stage during the follow‐up period. During the follow‐up period, there was no infection, loosening of internal fixation, fracture displacement, and osteonecrosis of femoral head. This depends on the accurate localization and reliable fixation of the three cannulated screws during the operation. Of course, long‐term complications, such as osteonecrosis of the femoral head, need to be followed up and observed for a longer period of time. This will be supplemented and improved in further research in the future. Cannulated screw internal fixation assisted by the orthopaedic surgery robot positioning system is a new surgical method for the treatment of femoral neck fractures. The results show that the new method can effectively shorten the operation time, reduce the times of intraoperative fluoroscopy, reduce the amount of blood loss, accurately locate the placement direction of the screw guide needles, and improve the success rate of one‐time screw placement. It is, therefore, beneficial to the early recovery of patients. The Harris hip score after operation was higher than that of the traditional operation. The recovery of hip joint function is better, which can significantly improve the curative effect.

The use of the orthopaedic surgery robot positioning system assisted cannulated screw internal fixation in the treatment of femoral neck fractures is widely applicable. As long as it can meet the requirements of closed reduction and internal fixation, this method can be used in patients with femoral neck fractures. This surgical method has the following application advantages: (i) the layout of the equipment is simple, it does not affect the original equipment layout in the operating room, and there is no need to consider device occlusion; (ii) the process of operation is smooth, and the operation is programmed. After successful closed reduction of femoral neck fractures, it is only necessary to use the C‐arm X‐ray machine to collect the positive and lateral images of the affected hip joint. According to the software system prompt, we can plan the path of three screws at a time in the workstation. The robotic arm runs independently one by one; (iii) placement of screw guide needle is accurate. The robotic arm of orthopaedic surgery robot can move to the planned position accurately, ensuring that the surgical path is consistent with the planned path. The guide needle is in place in the process of real‐time monitoring and automatic path correction, effectively ensuring the safety of the operation and assisting the surgeon to complete the implantation; (iv) the operation is minimally invasive, and the surgical trauma is small. It is helpful to promote the rapid recovery of patients after operation. The traditional surgical method is to expose the lateral wall of the femur through an incision before the guide needle is placed. In the process, we need to cut the skin, subcutaneous tissue and broad fascia, ensuring pure separation of the lateral thigh muscle. Compared with the traditional operation method, the robotic arm is operated with the aid of the orthopaedic surgery robot positioning system. Follow the planned path navigation to locate the percutaneous placement of the guide needle. There is no need to make a long incision to expose the neck of the thigh. There was no excessive soft tissue injury. The operative blood loss and the degree of surgical trauma were reduced. To make the operation safer and more effective, the minimally invasive operation was realized. It is, therefore, more conducive to fracture healing and early postoperative rehabilitation exercise. To some extent, it solves the limitation of the surgeon's visual field, reduces the fracture exposure area, and achieves the purpose of minimally invasive small incision. It also satisfies the concept of rapid recovery of surgery to a certain extent; and (v) the exposure time of radiation is less. With the aid of the orthopaedic surgery robot positioning system, the frequency of intraoperative fluoroscopy is reduced, and the operation time is shortened. It can effectively reduce the X‐ray damage of patients and medical staff. It therefore has a protective effect on patients and medical staff.

To sum up, cannulated screw internal fixation assisted by the orthopaedic surgery robot positioning system is an ideal method for the treatment of femoral neck fractures. The operation of orthopedic robot navigation equipment is relatively simple. To overcome the shortcomings of traditional surgical methods, such as unstable operation, visual deviation and so on, it can accurately locate the placement direction of screw guide needles, improve the success rate of one‐time screw placement, shorten the operation time, and reduce the surgical trauma. It can effectively reduce the X‐ray damage of patients and medical staff and promote the rapid recovery of patients after operation. In turn, the minimally invasive operation was realized. However, there are still some shortcomings to this study, as the number of cases collected is relatively small and needs to be improved in the future.

## References

[1] Kwoh YS , Hou J , Jonckheere EA , Hayati S . A robot with improved absolute positioning accuracy for CT guided stereotactic brain surgery. IEEE Trans Biomed Eng, 1988, 35: 153–160. 3280462 10.1109/10.1354

[2] Taylor RH , Mittelstadt BD , Paul HA , *et al* An image‐directed robotic system for precise orthopaedic surgery. IEEE Trans Robot Autom, 1994, 10: 261–275.

[3] Plaskos C , Cinquin P , Lavallée S , Hodgson AJ . Praxiteles: a miniature bone‐mounted robot for minimal access total knee arthroplasty. Int J Med Robot, 2005, 1: 67–79. 10.1002/rcs.59 17518407

[4] Ringel F , Stüer C , Reinke A , *et al* Accuracy of robot‐assisted placement of lumbar and sacral pedicle screws: a prospective randomized comparison to conventional freehand screw implantation. Spine, 2012, 37: 496–501. 10.1097/BRS.0b013e31824b7767 22310097

[5] Dreval ON , Rynkov IP , Kasparova KA , Bruskin A , Aleksandrovskiĭ V , Zil'bernshteĭn V . Results of using spine assist mazor in surgical treatment of spine disorders. Zh Vopr Neirokhir Im N N Burdenko, 2014, 78: 14–20. 25146652

[6] Schatlo B , Molliqaj G , Cuvinciuc V , Kotowski M , Schaller K , Tessitore E . Safety and accuracy of robot‐assisted versus fluoroscopy‐guided pedicle screw insertion for degenerative diseases of the lumbar spine: a matched cohort comparison. J Neurosurg Spine (Phila Pa 1976), 2014, 20: 636–643. 24725180 10.3171/2014.3.SPINE13714

[7] Grossman DC , Curry SJ , Owens DK , *et al* Vitamin D, calcium, or combined supplementation for the primary prevention of fractures in community‐dwelling adults: us preventive services task force recommendation statement. JAMA, 2018, 319: 1592–1599. 29677309 10.1001/jama.2018.3185

[8] US preventive services task force , Curry Susan J , Krist Alex H , *et al* Screening for osteoporosis to prevent fractures: us preventive services task force recommendation statement. JAMA, 2018, 319: 2521–2531. 29946735 10.1001/jama.2018.7498

[9] Florsehutz AV , Langford JR , Haidukewych G , Koval KJ . Femoral neck fractures: current management. J Orthop Trauma, 2015, 29: 121–129. 25635363 10.1097/BOT.0000000000000291

[10] Thillemann TM , Pedersen AB , Johnsen SP , Søballe K , Danish Hip Arthroplasty Registry . Implant survival after primary total hip arthroplasty due to childhood hip disorders results from the danish hip arthroplasty registry. Acta Orthop, 2016, 87: 567–569. 19085493 10.1080/17453670810016830

[11] Vaishya R , Agarwal AK , Gupta N , Vijay V . Sartorius muscle pedicleiliac bone graft for the treatment of avascular necrosis of femur head. J Hip Preserv Surg, 2016, 3: 215–222. 27583161 10.1093/jhps/hnw012 PMC5005060

[12] Bernadeue D , Bauke K , Tania F , Mohit B . Decision making: open reduction internal fixation versus arthroplasty for femoral neck fractures. Technol Orthop, 2008, 23: 288–295.

[13] Schep NW , Heintjes RJ , Martens EP , van Dortmont LM , van Vugt AB . Retrospective analysis of factors influencing the operative result after percutaneous osteosynthesis of intracapsular femoral neck fracrures. Injury, 2004, 35: 1003–1009. 15351667 10.1016/j.injury.2003.07.001

[14] Raaymakers EL . Fractures of the femoral neck: a review and personal statement. Acta Chir Orthop Traumatol Cech, 2006, 73: 45–59. 16613748

[15] Wu XB , Wang JQ , Sun X , Han W . Guidance for the treatment of femoral neck fracture with precise minimally invasive internal fixation based on the orthopaedic surgery robot positioning system. Orthop Surg, 2019, 11: 335–340. 31062519 10.1111/os.12451 PMC6595117

[16] Leung KS , Tang N , Cheung LW , Ng E . Image‐guided navigation in orthopaedictrauma. J Bone Joint Surg Br, 2010, 92: 1332–1337. 20884967 10.1302/0301-620X.92B10.24594

[17] Pauyo T , Drager J , Albers A , Harvey EJ . Management of femoral neck fractures in the young patient: a critical analysis review. World J Orthop, 2014, 5: 204–217. 25035822 10.5312/wjo.v5.i3.204 PMC4095012

[18] Forsh DA , Ferguson TA . Contemporary management of femoral neck fractures: the young and the old. Curr Rev Musculoskelet Med, 2012, 5: 214–221. 22628175 10.1007/s12178-012-9127-x PMC3535087

[19] Oakey JW , Stover MD , Summers HD , Sartori M , Havey RM , Patwardhan AG . Does screw configuration affect subtrochanteric fracture after femoral neck fixation. Clin Orthop Relat Res, 2006, 443: 302–306. 16462455 10.1097/01.blo.0000188557.65387.fc

[20] Gjertsen JE , Vinje T , Engesaeter LB , *et al* Internal screw fixation compared with bipolar hemiarthroplasty for treatment of displaced femoral neck fractures in elderly patients. J Bone Joint Surg Am, 2010, 92: 619628. 10.2106/JBJS.H.01750 20194320

[21] Wang JQ , Sun L , Wang MY . Application and progress in computer assisted orthopaedic surgery. Front Surg, 2004, 6: 110–114.

